# *Operando* Visualization of Cathode Water Management in Proton Exchange Membrane Water Electrolyzers Using Synchrotron X‐Ray Radiography

**DOI:** 10.1002/advs.77246

**Published:** 2026-08-19

**Authors:** Wataru Yoshimune, Tetsuichiro Hayakawa, Yuta Miyase

**Affiliations:** ^1^ Toyota Central R&D Labs., Inc. Nagakute Aichi Japan

**Keywords:** hydrogen crossover, *operando* synchrotron X‐ray radiography, proton exchange membrane water electrolyzers, water management, water vapor carryover

## Abstract

Optimizing water management in proton exchange membrane water electrolyzers (PEMWEs) is essential for reducing the levelized cost of hydrogen and ensuring safe operation, as it influences both water vapor carryover and hydrogen crossover. In this study, we establish an *operando* synchrotron X‐ray radiography system to visualize the liquid water distribution and dynamics within the cathode gas diffusion layer. Quantitative analysis of the time‐resolved radiographic data reveals that the cathode water behavior is primarily governed by current density through its effect on electro‐osmotic water supply and hydrogen‐gas‐driven water removal, together with cell temperature through liquid‐vapor phase transitions. At lower temperatures, liquid water preferentially accumulates beneath the flow‐field ribs due to localized current density and limited evaporative drying. Increasing temperature enhances evaporation, suppresses liquid water accumulation, and shifts the dominant transport mode from continuous to pulsating water transport. The results indicate that evaporation‐condensation processes play a critical role in regulating cathode water dynamics. These findings provide mechanistic insight into cathode water management and its implications for water vapor carryover and hydrogen crossover in PEMWE systems.

## Graphical abstract

The cathode of proton exchange membrane water electrolyzers remains a poorly understood region despite its importance for hydrogen purity and operational safety. *Operando* synchrotron X‐ray radiography directly visualizes liquid water behavior and uncovers a transition from continuous to pulsating transport, providing new insight into multiphase transport processes relevant to next‐generation green hydrogen technologies.

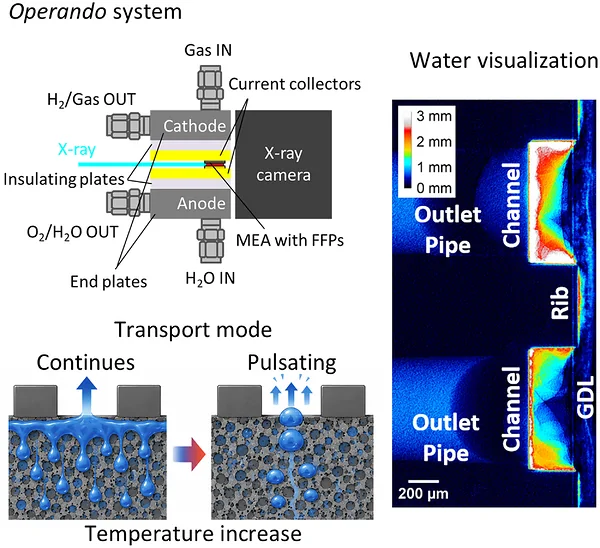

## 1 Introduction

The global transition toward net‐zero emissions by 2050 is accelerating the deployment of renewable energy technologies [1]. Wind and solar power resources are widely used, but their electrical output is inherently intermittent because it depends on weather conditions and the time of day. In addition, electricity supply often peaks around midday, whereas demand reaches its maximum at sunset [2]. This mismatch between supply and demand poses a serious challenge to power operations.

Hydrogen is attracting attention as an effective means of large‐scale energy storage to address the temporal mismatch. Global hydrogen demand has already reached about 100 million tons in 2024 [3]. It is expected to increase to 300–400 million tons by 2050 [4], implying that terawatt‐scale water electrolysis capacity will be required [5]. To make green hydrogen competitive with conventional steam reforming, a levelized cost of hydrogen (LCOH) below 2 USD kg_H2_^−1^ has been proposed as an important target [6].

The primary water electrolysis systems used for large‐scale hydrogen production from renewable electricity are alkaline water electrolysis (AWE) [7] and proton exchange membrane water electrolysis (PEMWE) [8]. AWE systems are technologically mature and suitable for large installations. However, they are not necessarily optimal for future requirements such as rapid response to fluctuating renewable power, frequent startup and shutdown, compact system design, and high‐pressure operation. PEMWE systems offer fast dynamic response, stable operation at low load, and the possibility of producing hydrogen at high pressure, making them attractive for coupling with renewable energy sources.

Despite these advantages, two major challenges remain for the widespread development of PEMWEs: reducing the LCOH and ensuring operational safety. From the cost perspective, the use of iridium oxide at the anode and the energy consumption associated with auxiliary components remain major contributors to the LCOH [9]. From the safety perspective, hydrogen crossover through the membrane limits the use of thinner, high‐performance membranes and constrains operating conditions.

Cost reduction has therefore become a major focus of PEMWE development. The New Energy and Industrial Technology Development Organization (NEDO) has presented a roadmap for reducing LCOH with PEMWEs toward 2040 [10]. Assuming an electricity price of 2.5 JPY kW h^−1^, a capacity factor of 40%, a cell voltage of 1.8 V at 4.0 A cm^−2^, and a stack lifetime of 90 000 h, cost analysis suggests that reducing the iridium loading from the current value of 0.4 to 0.1 mg_Ir_ cm^−2^ could lower the LCOH to 18 JPY N^−1^ m^−3^ corresponding to about 1 USD kg_H2_^−1^. Furthermore, Hancke and co‐workers [11] performed a model study of a 10 MW PEMWE system and reported that balance‐of‐plant energy consumption accounts for roughly 10% of the total energy demand. Among these auxiliary systems, hydrogen drying is important because water vapor remains as an impurity in the produced hydrogen and must be removed before utilization. Current safety regulations in Japan limit the hydrogen outlet pressure to 1 MPa, whereas PEMWE systems are commonly operated at 3 MPa in other regions [10]. Because the water vapor content of saturated hydrogen decreases with increasing pressure, hydrogen at 3 MPa contains about 11.8 g kg_H2_^−1^ of water vapor compared with about 35.4 g kg_H2_^−1^ at 1 MPa. As a result, reducing the operating pressure from 3 to 1 MPa increases the energy required for hydrogen drying from 0.7 to 2.1 kW h kg_H2_^−1^. In addition, reducing water vapor content also decreases dryer size, regeneration frequency, corrosion risk in downstream components, and operational instability during dynamic load changes. Therefore, alongside efforts to reduce iridium loading, suppressing water vapor carryover from the PEMWE stack is an important strategy for reducing operating costs and improving system reliability.

Beyond its impact on drying costs, water transport through the membrane and subsequent water behavior at the cathode may also influence hydrogen crossover. Previous studies have suggested the possibility that liquid water accumulated at the cathode affects hydrogen crossover by acting as an additional transport barrier [12]. Therefore, cathode water management represents a common link between drying costs and hydrogen crossover, connecting both economic and safety considerations.

Previous visualization studies of liquid water in PEMWEs have mainly focused on oxygen bubble distribution within the anode porous transport layer (PTL) because of its impact on concentration overpotential [13]. Since the anode titanium‐based PTL strongly attenuates X‐rays, high photon energies, typically above 30 keV, are required for penetration. However, at such energies, the contrast between oxygen bubbles and water electrolytes becomes small, making direct bubble observation challenging [14, 15]. Consequently, recent research has concentrated on advances in bubble visualization techniques and analysis of the dynamics [16, 17, 18, 19, 20, 21, 22, 23]. Alternatively, neutron imaging has been employed to overcome these limitations in transmittance [24, 25, 26, 27, 28, 29, 30, 31, 32].

In contrast, the cathode gas diffusion layer (GDL) is composed of carbon‐based materials and can be readily penetrated by low‐energy X‐rays. *Operando* synchrotron X‐ray imaging therefore offers a unique opportunity to visualize liquid water within the cathode of PEMWEs. Although many studies have reported liquid water observations in the GDL of proton exchange membrane fuel cells (PEMFCs) [33, 34, 35, 36, 37, 38], which employ similar carbon‐based GDL materials, systematic investigations of liquid water distribution and dynamics within the cathode GDL of PEMWEs remain scarce.

This lack of understanding is particularly important because cathode water behavior governs how water is removed from the PEMWE system and may influence LCOH and operational safety. Water transported through the membrane enters the cathode catalyst layer (CL) and GDL, where it can either evaporate and leave with the hydrogen stream or accumulate as liquid water. The balance between these vapor‐phase and liquid‐phase pathways determines both the amount of water carried out with the produced hydrogen and the extent of liquid water accumulation within the cathode. Increased water vapor carryover raises the hydrogen drying requirement, whereas liquid water accumulation may influence hydrogen crossover. Therefore, understanding liquid water distribution and dynamics within the cathode GDL is essential for developing water management strategies that simultaneously address drying costs and hydrogen crossover.

In this study, we established an *operando* synchrotron X‐ray radiography system to investigate liquid water behavior within the cathode GDL of PEMWEs. We systematically examined the effects of cell temperature, current density, and cathode gas conditions on liquid water distribution and dynamics. Moreover, we discussed the mechanisms governing water removal from the cathode and their implications for water vapor carryover and hydrogen crossover.

## 2 Results and Discussion

### 2.1 *Operando* PEMWE System and Electrolysis Performance

An *operando* PEMWE cell was designed based on our previously developed PEMFC *operando* platform [39, 40, 41] to enable direct visualization of liquid water at the cathode (Figure 1a). The cell consisted of a membrane electrode assembly (MEA), in which an in‐house fabricated catalyst‐coated membrane (CCM) was sandwiched between a PTL and a GDL, and was sealed with different flow field plates (FFPs). The resulting *operando* cell had a geometric area of 0.24 cm^2^. To ensure both electrochemical stability and X‐ray transmittance, different materials were employed for the FFPs and gaskets on each electrode. A graphite‐based FFP with a 150 µm‐thick ethylene‐propylene‐diene‐monomer gasket was used at the cathode to facilitate X‐ray visualization of liquid water [39], while a metallic FFP with a 200 µm‐thick polytetrafluoroethylene gasket was used at the anode to mitigate carbon corrosion under high‐voltage conditions [41]. The gasket thicknesses were selected to define the compression ratio of the PTL and GDL, respectively. With this choice of FFPs and gaskets, liquid water was observable only at the cathode in this setup (Figure 1b). Both FFPs were designed with three straight channels (rib width: 0.6 mm, channel width: 0.55 mm, depth: 0.33 mm). Due to the limited field of view, the focus was maintained on two channels and the intervening rib. In this work, water was supplied at a rate of 0.35 mL min^−1^ from the anode inlet (Figure S1).

**FIGURE 1:**
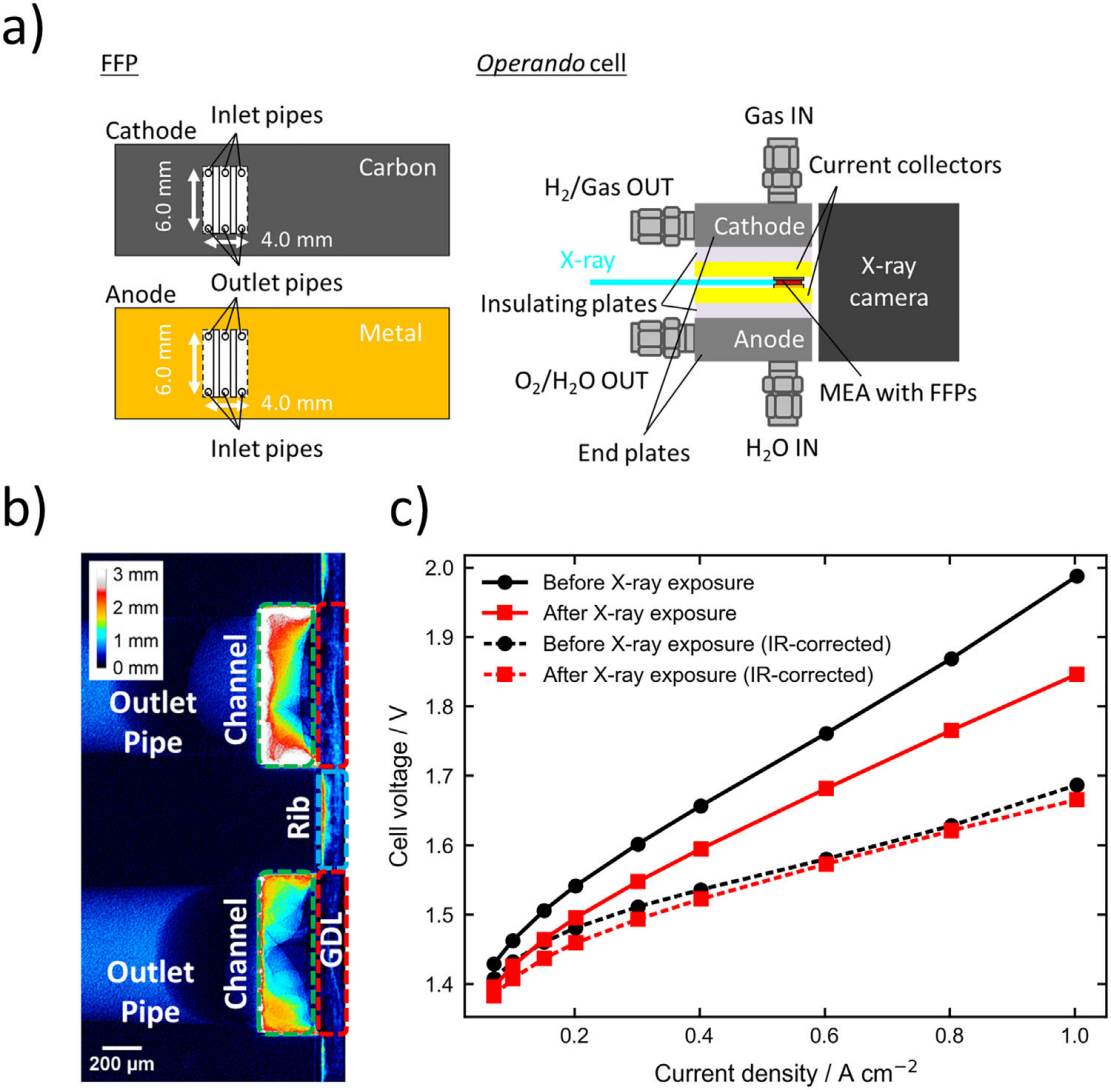
(a) Configuration of the *operando* PEMWE cell: (left) top view of the cathode (graphite‐based) and anode (metallic) FFPs; (right) schematic of the experimental setup for synchrotron X‐ray radiography. (b) Typical differential X‐ray radiograph showing liquid water distribution (color‐coded for water thickness) within the cathode gas channels and GDL. The dashed outlines indicate the regions of interest used for the quantitative analysis. (c) Polarization curve at 50°C and ambient pressure measured before (black) and after (red) 4 h of continuous X‐ray exposure. Solid lines represent the measured cell voltage, while dashed lines indicate the IR‐corrected voltage based on HFR.

Figure 1c shows the polarization curves at 50°C measured before and after 4 h of X‐ray exposure. Prior to X‐ray irradiation, a current density of 1 A cm^−2^ was achieved at a cell voltage below 2 V. After prolonged X‐ray exposure, a decrease in cell voltage was observed, indicating an apparent improvement in electrolysis performance. This change is consistent with the IR‐corrected curves based on high‐frequency resistance (HFR), suggesting that the apparent enhancement originated from an approximately 50% reduction in membrane resistance induced by X‐ray irradiation. A similar trend has been reported for PEMFCs [42], where membrane resistance decreased after several hours of X‐ray exposure. In PEMFCs, long‐term X‐ray irradiation typically leads to performance degradation due to a loss of oxygen reduction reaction activity [43]. In contrast, in the present PEMWE system, most incident X‐rays were attenuated at the anode by the metallic FFP, which likely suppressed the degradation of oxygen evolution reaction (OER) activity. Although the hydrogen evolution reaction (HER) activity at the cathode might be affected, the overall cell performance, which was measured as the potential difference between the electrodes, was primarily governed by OER kinetics. Therefore, the influence of HER degradation was negligible, and the observed performance change can be attributed mainly to the reduction in membrane resistance. Although the present experiments were performed under galvanostatic conditions, where the reaction rate was fixed by the applied current density, the X‐ray‐induced reduction in membrane resistance decreased the cell overpotential and may therefore have altered the heat generation within the cell. Consequently, the influence of prolonged X‐ray irradiation on the observed water behavior cannot be completely excluded and should be considered when interpreting the temperature dependence reported in this study. Additional experimental considerations relevant to the quantitative interpretation of the present results are summarized in Section S2.

The temporal evolution of cell voltage during constant‐current operation at 1 A cm^−2^ under X‐ray exposure is displayed in Figure S2. Regardless of X‐ray irradiation, transient voltage fluctuations were observed immediately after the start of the stability test. Under continuous X‐ray exposure, these fluctuations gradually stabilized within approximately 2 h, accompanied by a decrease in overpotential. This decrease was attributed to the reduction in membrane resistance discussed above. The specific mechanism responsible for the stabilization of voltage fluctuations following the overpotential decrease was not identified in this study.

Although PEMWEs are typically operated without cathode gas flow, dry or humidified gas was also supplied to the cathode in selected experiments to systematically control water removal conditions and investigate the roles of evaporation. The experimental conditions at open circuit voltage (OCV) and during electrolysis operation (0.25, 0.5, 0.75 and 1.00 A cm^−2^) for imaging are summarized in Table S1, including cell temperature, cathode gas flow rate and gas humidity as control parameters. Following the imaging protocols (Figure S3), the cell was operated at 50°C, 60°C and 70°C under five cathode gas conditions: no flow, dry gas flow (20 and 40 mL min^−1^), and wet gas flow (80% relative humidity, 20 and 40 mL min^−1^). Figure S4 summarizes the electrolysis performance during image acquisition. The absence of data at 1.00 A cm^−2^ (60°C and 70°C) and 0.75 A cm^−2^ (70°C) under dry gas flow indicates that the cell voltage reached the cutoff cell voltage (2.5 V) due to membrane dehydration.

### 2.2 Water Saturation

The time‐averaged liquid holdup in the gas channel and water saturation in the GDL beneath the ribs and beneath the channels were analyzed, with the results summarized in Figure 2. This systematic analysis revealed that the water distribution at the cathode was governed by three key parameters: (i) cell temperature, (ii) gas condition, and (iii) current density.

**FIGURE 2:**
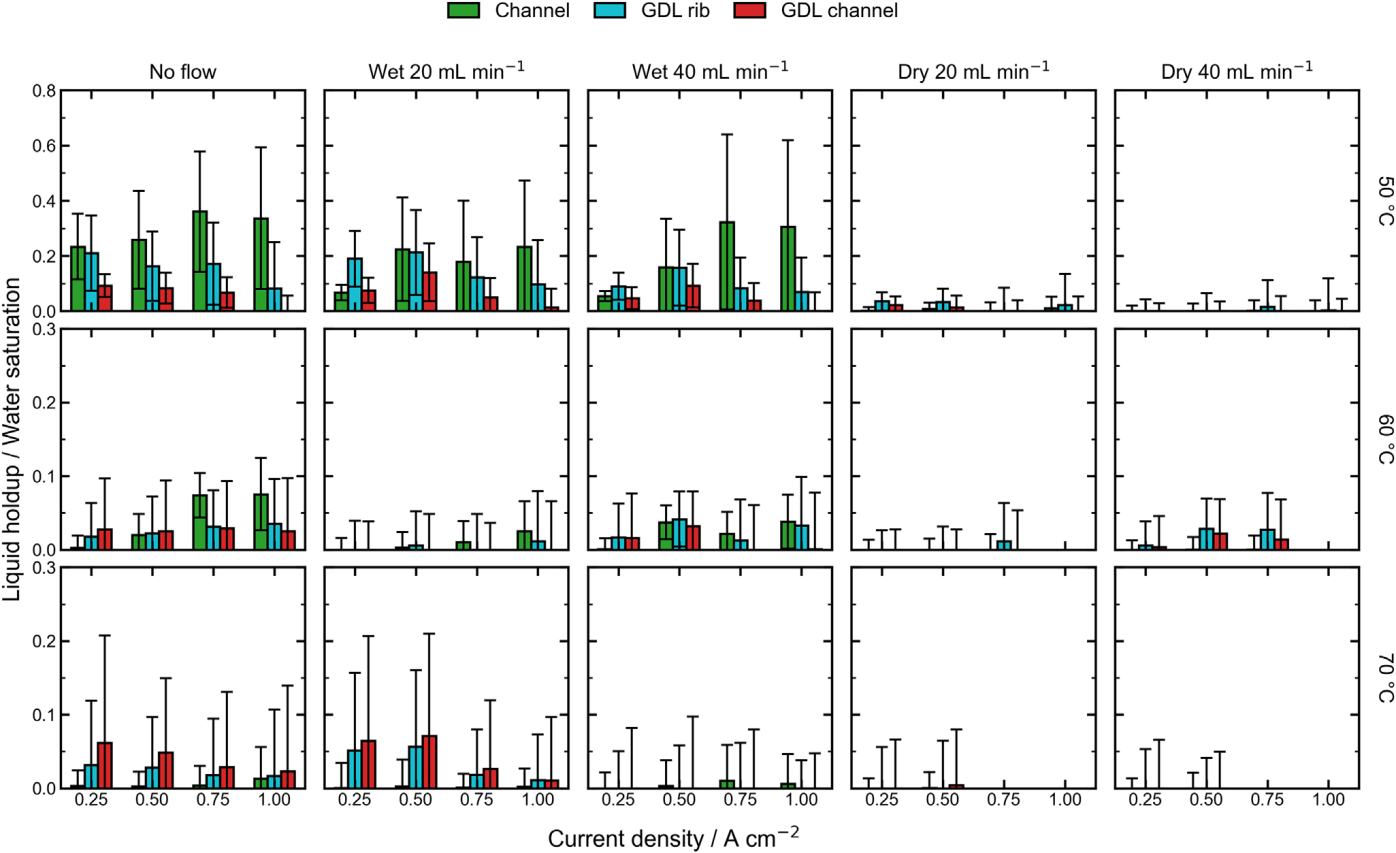
Average liquid holdup within the cathode gas channels (green) and water saturation in the GDL beneath the ribs (cyan) and channels (red) as a function of current density. The data are presented for cell temperatures (50°C, 60°C, and 70°C; rows) and cathode gas flow conditions (no flow, wet gas, and dry gas at 20 or 40 mL min^−1^; columns). The data are presented as mean ± standard deviation (SD) calculated from the time‐series data acquired during steady‐state operation. The error bars represent the temporal standard deviation. Each operating condition was measured once (*n* = 1). No statistical hypothesis testing was performed.

The water saturation values shown in Figure 2 are calculated using the nominal porosity of the uncompressed GDL. Consequently, the absolute saturation values may be underestimated because the local pore volume decreases under compression, particularly beneath the ribs, where the compression ratio is approximately 30%. In addition, the present radiography provides only thickness‐integrated radiographs, and the local pore volume within the carbon paper and microporous layer (MPL) cannot be accurately determined from these images. Therefore, the equivalent water thickness shown in Figure S5 is provided as a complementary quantity that is independent of assumptions regarding the GDL porosity.

With increasing cell temperature (from top to bottom in Figure 2), both the liquid holdup in the gas channels and the water saturation in the GDL decreased. This is likely due to the increase in saturated vapor pressure, which is approximately 2.4 times higher at 70°C compared to 50°C. Consequently, at higher temperatures, most of the water transported via electro‐osmotic drag evaporated and was removed in the vapor phase. Remarkably, the dominant location of water accumulation in the GDL shifted from beneath the ribs to beneath the channels as the temperature increased. Under cell compression, the local current density is higher beneath the ribs, leading to localized hydrogen evolution and a corresponding influx of electro‐osmotic water. Accordingly, water accumulation preferentially occurs in the GDL beneath the ribs. At lower temperatures, water is more likely to condense within the GDL near the ribs of FFPs. This results in higher water saturation in the GDL beneath the ribs. Conversely, at higher temperatures, water saturation is lower beneath the ribs than in the channels because evaporation is accelerated beneath the ribs, where the produced hydrogen gas is concentrated.

Gas flow conditions also influenced the removal of accumulated water in the GDL through both shear‐induced drainage and evaporative drying. Within the investigated operating range, the effect of the gas flow rate was relatively minor compared to the impact of gas humidity (from left to right in Figure 2), indicating that evaporative transport dominated over shear‐induced drainage under these conditions. This vapor‐phase removal is even more pronounced for water accumulated within the gas channels.

With respect to current density (horizontal axis in each plot of Figure 2), contrasting trends were observed between the gas channels and the GDL. The amount of electro‐osmotic water is proportional to the current density, suggesting a monotonic increase in water saturation. This intuitive trend was only observed for the liquid holdup in the gas channels. In the GDL, the opposite trend occurred. This discrepancy can be explained by the removal of liquid water facilitated by the produced hydrogen gas. The velocity of the hydrogen gas increases linearly with current density, enhancing the removal of liquid water from the GDL pores (Figure 3). This result highlights the competing effects of current density on cathode water behavior: electro‐osmotic drag increases water supply to the cathode, whereas the accompanying increase in hydrogen‐gas flux enhances water removal from the GDL. Under the present conditions, the latter effect dominated within the GDL. However, this gas‐driven removal was less effective in the gas channels. At higher gas velocities, the flow regime transitions from slug to annular flow, where liquid water tends to persist as a thin film along the channel walls. Indeed, this regime shift was clearly observed within the outlet pipes (Figure 3), resulting in an observed increase in the liquid holdup within the channels at higher current densities.

**FIGURE 3:**
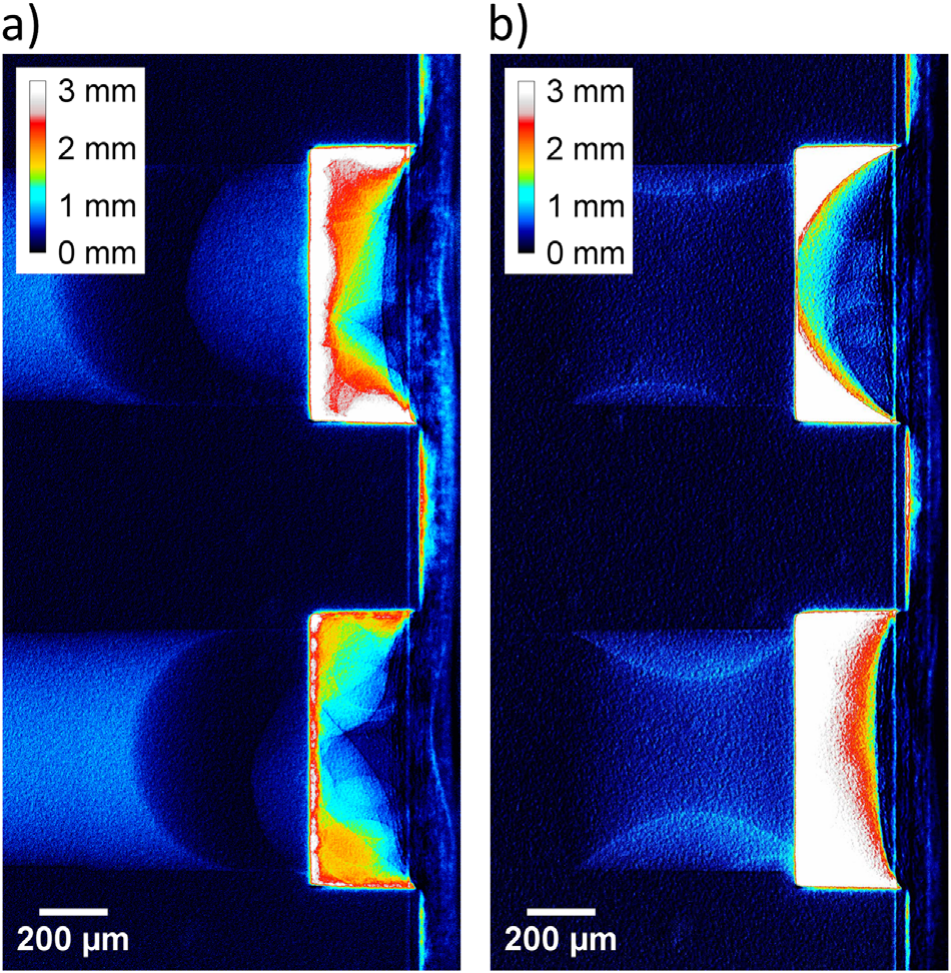
Representative snapshots of liquid water distribution at 50°C under no flow conditions at current densities of (a) 0.50 A cm^−2^ and (b) 1.00 A cm^−2^. The color scale indicates the water thickness, and the scale bars represent 200 µm.

### 2.3 Water Dynamics

The large error bars in the time‐averaged water saturation presented in Figure 2 indicate that the water saturation fluctuated even under steady‐state operation. In this subsection, the dynamics of liquid water within the gas channels and the GDL are discussed. Figure 4 shows the time evolution of water saturation during electrolysis at 50°C, 60°C, and 70°C under no flow conditions. The results for all experiments are presented in Figures S6–S8 and Movies S1–S5. Distinct differences in temporal behavior were observed between 50°C and 60°C–70°C.

**FIGURE 4:**
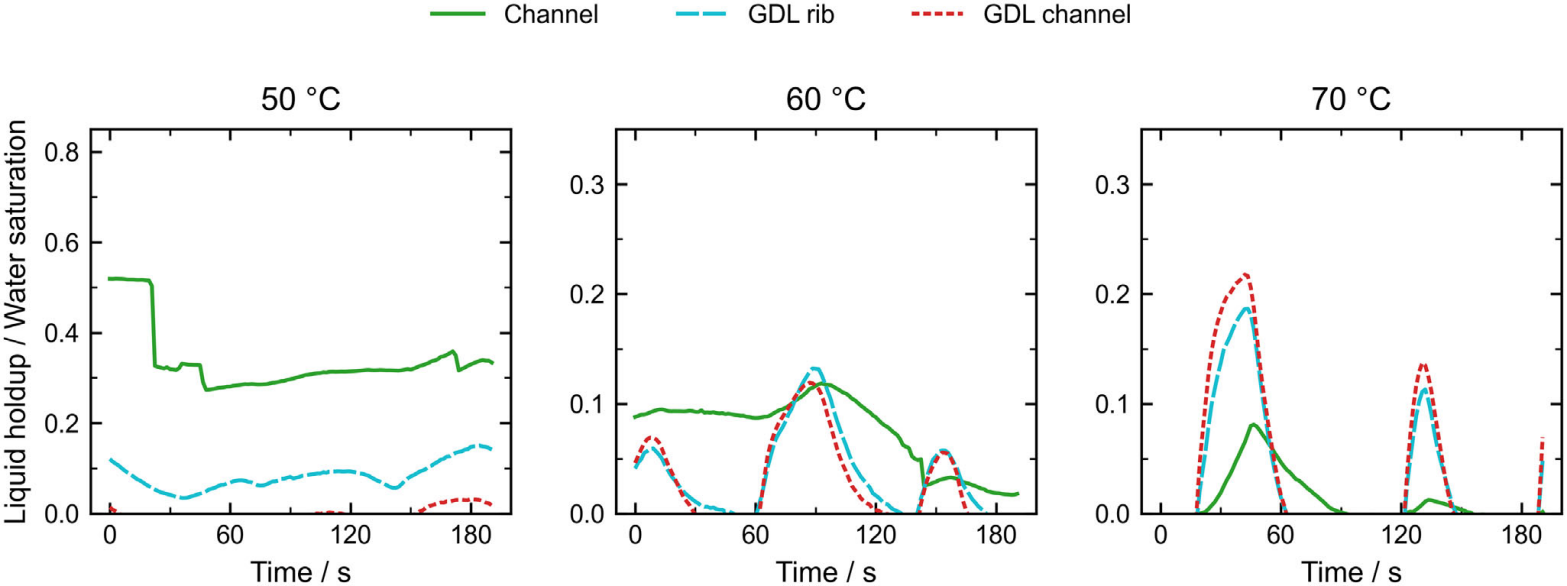
Time evolution of water accumulation at a current density of 1.00 A cm^−2^ under no flow conditions at (left) 50°C, (center) 60°C, and (right) 70°C. The green solid, cyan dashed, and red dotted lines represent the liquid holdup in the gas channels and the water saturation in GDL beneath the ribs/channels, respectively.

At 50°C, the water saturation in the GDL exhibited gradual and continuous variations over time. In the gas channels, intermittent drops in water saturation were observed, which is indicative of the presence of slug flow with intermittent water discharge. In contrast, at 60°C and 70°C, a pronounced pulsating behavior was observed in both the gas channels and GDLs. Quantitative analysis using a temporal autocorrelation function revealed a characteristic time periodicity of approximately 10–30 s for these pulsations. This timescale is consistent with a dynamic balance between the water storage capacity at the cathode (Section S8) and the water accumulation rate originating from electro‐osmotic drag. Furthermore, Figure S9 shows the results of calculating the pulsation rate, defined as the average of accumulation and discharge rates. The pulsation rate was higher at 70°C than at 60°C (approximately twice as fast), indicating that the intensity of pulsation was temperature dependent.

Notably, the pulsating behavior was only weakly affected by cathode gas flow conditions (Figures S6–S8), despite the significant influence of gas flow and humidity on the average water saturation shown in Figure 2. This contrast suggests that evaporative drying influenced the overall liquid water saturation within the GDL, whereas the pulsation rate was governed by temperature.

Based on the differential radiographs acquired at 50°C and 70°C, Figure 5 schematically illustrates the inferred liquid water transport pathways within the cathode GDL. At lower temperatures, liquid water was distributed over a broad region within the GDL, forming continuous liquid water pathways (Figure 5a). These pathways allowed liquid water to be removed continuously toward the gas channel. In contrast, at higher temperatures, liquid water became more localized owing to enhanced evaporative drying, and the continuous liquid water pathways observed at lower temperatures became discontinuous (Figure 5b). Consequently, liquid water likely accumulated locally near the CL/MPL interface and was released intermittently toward the gas channel.

**FIGURE 5:**
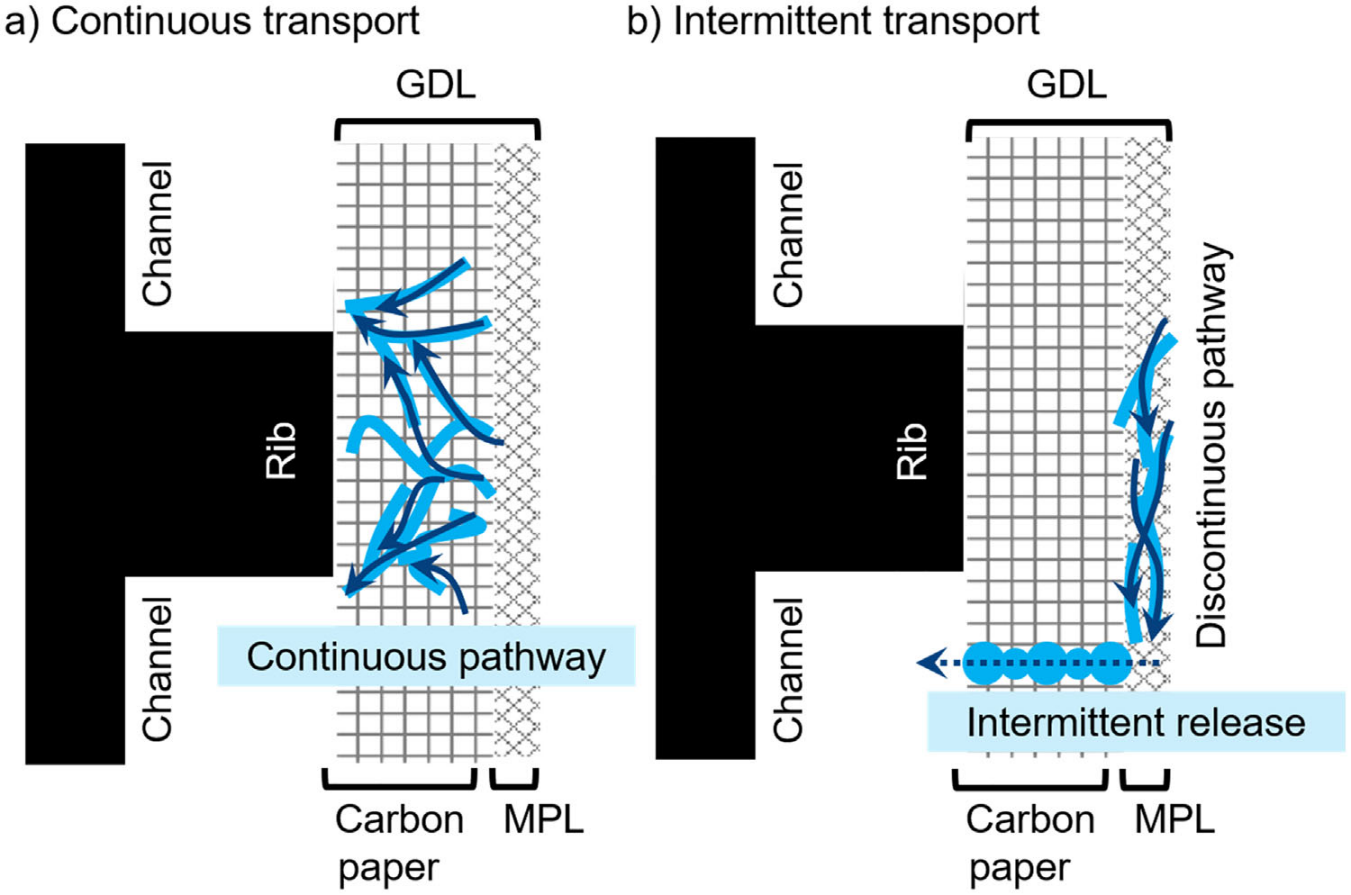
Schematic illustration of the inferred water transport pathways based on the observed liquid water distributions and temporal behavior. (a) At lower temperatures, continuous liquid water pathways are formed within the GDL, enabling continuous water transport toward the gas channels. (b) At higher temperatures, the continuous pathways become discontinuous owing to enhanced evaporation, and liquid water is released intermittently toward the gas channels.

These observations indicate that the transition from continuous to pulsating water dynamics originated from a temperature‐dependent change in the dominant water‐transport pathway. At lower temperatures, evaporation was followed by condensation within the GDL, promoting continuous liquid water transport. At higher temperatures, suppressed condensation likely disrupted continuous liquid water transport pathways, forcing water removal to proceed through intermittent accumulation‐release cycles.

### 2.4 Perspectives on Reducing Cathode Drying Costs and Mitigating Hydrogen Crossover

The discussion before this subsection has focused on the liquid water distribution and dynamics directly observed via *operando* synchrotron X‐ray radiography. In this subsection, we discuss the possible implications of these observations for water vapor carryover and hydrogen crossover.

The produced hydrogen gas carries water vapor generated by evaporation at the cathode. In the gas channels and piping, liquid water primarily adheres to the walls, meaning the gas/liquid interfacial area is limited. Consequently, both the effective liquid water surface area and the gas transport resistance within the cathode GDL are expected to influence the water vapor content of the produced hydrogen. In the present study, the water saturation within the GDL remained below approximately 0.2 beneath both the ribs and channels. Even when empirical correlations for the effective gas diffusion coefficient commonly used in PEMFC research are applied [44, 45, 46], the observed liquid water saturation corresponds to only a moderate reduction (∼30%) in gas diffusivity compared with a dry state. The present observations suggest that neither bulk liquid water accumulation within the GDL nor a severe reduction in hydrogen diffusivity is likely to dominate the water vapor content of the produced hydrogen gas. Instead, the effective gas/liquid interfacial area associated with microscopic liquid water morphology is expected to play a more important role in determining water evaporation and subsequent vapor carryover. However, the present two‐dimensional radiographic technique cannot directly quantify droplet morphology or interfacial surface area, highlighting the need for future three‐dimensional analysis using synchrotron X‐ray tomography. Nevertheless, the finding that excessive liquid water accumulation is absent under the investigated conditions provides useful insight into cathode water management strategies for reducing drying requirements.

Hydrogen crossover has been proposed to proceed via dissolution of hydrogen into liquid water at the cathode, followed by diffusion through the proton exchange membrane (PEM) and subsequent release into the anode compartment [47]. Although previous studies have reported that hydrogen crossover increased with operating temperature and attributed this behavior to enlargement of water clusters within the membrane [48], the dominant mechanism remains under debate. The intermittent water discharge behavior observed at 60°C –70°C in the present study may provide an additional perspective on this issue. The present observations raise the possibility that liquid water is temporarily retained near the CL/MPL interface by the capillary barrier and is released intermittently once the capillary pressure threshold is exceeded. Hydrogen generation continues while water is retained near the CL/MPL interface, potentially leading to an increase in the local hydrogen partial pressure. According to Henry's law, the solubility of hydrogen in liquid water increases with hydrogen partial pressure, which may enhance hydrogen crossover. Interestingly, a recent study has reported that replacing a hydrophobic MPL with a hydrophilic MPL suppressed hydrogen crossover [12]. Although direct evidence linking pulsation behavior to hydrogen crossover is not yet available, the present observations suggest that water storage dynamics near the CL/MPL interface, in addition to membrane properties alone, may influence hydrogen crossover. These observations suggest that cathode water management may play a role not only in controlling water vapor carryover but also in mitigating hydrogen crossover.

## 3 Conclusion

We successfully visualized the liquid water distribution and dynamics within the cathode GDL of a PEMWE using *operando* synchrotron X‐ray radiography. The results revealed that cathode water behavior is governed by the interplay between electro‐osmotic water supply, hydrogen‐gas‐driven water removal, and temperature‐dependent liquid–vapor phase transitions. The present observations provide new insight into the mechanisms governing cathode water removal in PEMWEs. In particular, the results highlight the importance of evaporation‐condensation processes and CL/MPL interfacial water dynamics in controlling liquid‐water behavior. These findings contribute to a better understanding of cathode water behavior and provide perspectives on possible implications for water vapor carryover and hydrogen crossover. The *operando* methodology established in this work provides a useful platform for investigating cathode water behavior and generating mechanistic insights that can serve as design guidelines for future materials development, cell architecture optimization, and operating protocols in PEMWE systems.

## 4 Experimental Section

### 4.1 Materials

The CCM with a geometric area of 25 cm^2^ was fabricated by a spray‐coating method. A Nafion 117 membrane (N117, Chemours, USA) was used as the PEM. The anode CL consisted of IrO_2_ (SA‐5, Tanaka Kikinzoku Kogyo, Japan) with an ionomer‐to‐catalyst weight ratio of 0.2. The cathode CL featured a platinum‐supported carbon catalyst (Pt/C, TEC10E50E, Tanaka Kikinzoku Kogyo, Japan) with an ionomer‐to‐carbon weight ratio of 1.0. Nafion ionomer (DE2020, CS type, FUJIFILM Wako Pure Chemical, Japan) served as the binder and proton conductor in both CLs. On the anode side, a platinum‐coated titanium felt (Nikko Techno, Japan) was employed as the PTL, characterized by a fiber diameter of 20 µm, a thickness of 200 µm, and a porosity of 56%. On the cathode side, a carbon paper with an MPL was used as the GDL (GDS3260, Avcarb, Germany), featuring a total thickness of 210 µm and a porosity of 60%.

### 4.2 Electrochemical Setup and Test Protocols

The cell voltage and current density were controlled using a potentiostat/galvanostat (HZ‐7000, Hokuto Denko, Japan), while the high‐frequency resistance (10 kHz) was measured with an AC resistance meter (356E, Tsuruga Denki, Japan). The *operando* cell was operated at ambient pressure using a custom‐built fuel cell test bench (Chino, Japan). The electrochemical characterization protocol consisted of four steps: (i) break‐in, (ii) initial performance test, (iii) steady‐state operation for imaging, and (iv) final performance test. During the break‐in, the cell was maintained at OCV for 5 min after reaching 50°C, followed by conditioning at 1.9 V for 2 min [23]. Polarization curves were obtained by ramping the current density from 0.10 to 1.00 A cm^−2^ with a cutoff voltage of 2.5 V. Steady‐state operation was carried out at current densities of 0.25, 0.50, 0.75 and 1.00 A cm^−2^, ensuring the cell voltage remained below 2.5 V. For each steady‐state condition (Table S1), the cell was stabilized for 2 min at OCV and at these target current densities, followed by 3 min of X‐ray irradiation for imaging (Figure S3). Between conditions, the cathode was purged with dry nitrogen gas at 200 mL min^−1^ for 2 min to eliminate residual water [46]. After imaging, electrochemical degradation was evaluated by conducting the final performance test.

### 4.3 Synchrotron X‐Ray Radiography

*Operando* experiments were performed at the Toyota Beamline (BL33XU) of SPring‐8 in Japan [49]. The incident X‐ray energy was 11.4 keV using a liquid‐nitrogen cooled Si(111) double‐crystal monochromator. Higher‐order harmonics were suppressed by Rh‐coated double total‐reflection mirrors. The X‐rays transmitted through the *operando* PEMWE cell were detected using an imaging system consisting of a LuAG:Ce single‐crystal scintillator with a 10 µm thick phosphor screen, a custom‐made AA 50 imaging unit with a 5× objective lens, and a sensor (ORCA‐Flash 4.0, Hamamatsu Photonics, Japan). The field of view was 2.66 × 2.66 mm^2^ with a pixel resolution of 1.3 µm. The exposure time was 1 s with a 0.5 s interval between successive exposures.

### 4.4 Imaging Analysis

The water distribution in the gas channels and cathode GDL was visualized by subtracting a reference image acquired at OCV under the same experimental conditions (cell temperature, cathode gas flow rate, and gas humidity) from the images captured during electrolysis. The mass of accumulated liquid water was quantified from the resulting differential images on a pixel‐by‐pixel basis using the Beer–Lambert law, applying an attenuation coefficient of 0.344 mm^−1^ at 11.4 keV [43]. The pixel‐wise water thickness in the gas channels and in the GDL beneath the ribs and channels was spatially averaged to obtain representative water thickness values. These average values were subsequently converted to liquid holdup in the gas channels and water saturation in the GDL beneath the ribs/channels, respectively. The temporal evolution of the averaged water saturation (or liquid holdup) was analyzed using a temporal autocorrelation function to identify characteristic periodicity. Pulsation peaks were identified as local maxima in the time‐series data, and the accumulation and release rates were calculated from the average slopes during the increasing and decreasing phases of each pulsation cycle, respectively. The detailed time‐series analysis procedure has been reported previously [50].

### 4.5 Statistical Analysis

No statistical hypothesis testing was performed because the objective of this study was to characterize liquid water behavior under each operating condition rather than to compare independent experimental populations. For each operating condition, the representative water thickness, liquid holdup, and water saturation were obtained by averaging the time‐resolved data acquired during steady‐state operation. The error bars represent the temporal standard deviation (mean ± SD) calculated from the time‐series data. No data transformation, normalization, or outlier exclusion were applied. Each operating condition was measured once (*n* = 1); therefore, the reported standard deviations describe temporal fluctuations within a single experiment rather than reproducibility between independent experiments. Data processing and image analysis were performed using Python.

## Author Contributions

W. Y. designed the experimental concept, carried out all experiments, and performed data analysis. T. H. prepared the imaging setup and provided technical support for the *operando* synchrotron experiments. Y. M. proposed experimental ideas, provided materials, and contributed to the discussion of the results. W. Y. wrote the first draft. All authors contributed to the revision of the manuscript and approved the final version.

## Funding

This research received no external funding.

## Conflicts of Interest

The authors declare no conflicts of interest.

## Supporting information




**Supporting File 1**: advs77246‐sup‐0001‐SuppMat.docx.

**Supporting File 2**: advs77246‐sup‐0002‐MovieS1.mp4.

**Supporting File 3**: advs77246‐sup‐0003‐MovieS2.mp4.

**Supporting File 4**: advs77246‐sup‐0004‐MovieS3.mp4.

**Supporting File 5**: advs77246‐sup‐0005‐MovieS4.mp4.

**Supporting File 6**: advs77246‐sup‐0006‐MovieS5.mp4.

## Data Availability

The data that support the findings of this study are available on request from the corresponding author.
