# Supplementary material for: Operando Visualization of Cathode Water Management in Proton Exchange Membrane Water Electrolyzers Using Synchrotron X‐Ray Radiography
Source: Adv Sci (Weinh). 2026 Aug 19;13(62):e77246. doi: 10.1002/advs.77246 (PMC13488480; doi:10.1002/advs.77246)
Supplement: Supplementary file 1 [file ADVS-13-e77246-s001.docx]

Supporting Information

*Operando* Visualization of Cathode Water Management in Proton Exchange Membrane Water Electrolyzers Using Synchrotron X-ray Radiography

Wataru Yoshimune,* Tetsuichiro Hayakawa, and Yuta Miyase

W. Yoshimune, T. Hayakawa, Y. Miyase

Toyota Central R&D Labs., Inc., 41-1 Yokomichi, Nagakute, Aichi 480-1192, Japan
E-mail:yoshimune@mosk.tytlabs.co.jp

**S1. Water supply method**

In this study, water was supplied using a fuel cell setup (Figure S1). Humidified nitrogen gas was supplied to the anode side^[S1]^ and cooled to near room temperature before the anode inlet to facilitate condensation.

**Figure S1.** Our *operando* X-ray radiography setup.

**S2. Additional considerations for image analysis**

The present *operando* synchrotron X-ray radiography experiments were performed using a small-sized PEMWE cell. Therefore, several limitations should be considered when interpreting the quantitative results.

- N_2_ was used as the cathode gas instead of H_2_ to systematically control the gas flow rate and humidity. Consequently, the gas environment differs from that in practical PEMWE operation. Because H_2_ has a lower viscosity and higher water-vapor diffusivity than N_2_, vapor carryover may differ quantitatively from that in practical-sized systems. Furthermore, the externally supplied N_2_ flow rates do not necessarily correspond to the self-generated H_2_ flow rate during electrolysis, and the difference depends on the location of interest (e.g., upstream or downstream of the flow field).
- Possible changes in the wettability of the GDL induced by prolonged X-ray irradiation were not investigated in the present study and therefore cannot be completely excluded.
- The investigated cathode gas flow rates (20–40 mL min^−1^) represent a relatively narrow range of low-shear conditions. Higher gas flow rates were not examined because stable electrolysis operation could not be maintained under dry-gas conditions owing to severe membrane dehydration. Therefore, the conclusion that gas flow rate has a smaller influence than gas humidity should be interpreted within the investigated operating range and should not be directly extrapolated to higher-shear conditions relevant to practical PEMWE systems.

**S3. X-ray damage**

In the past X-ray imaging studies on proton exchange membrane fuel cells (PEMFCs), X-ray irradiation damage was observed during prolonged X-ray imaging.^[S2,S3]^ In this work, we evaluated electrochemical stability of our proton exchange membrane water electrolyzers (PEMWE) system with and without X-ray irradiation (Figure S2).

**Figure S2.** The cell voltage stability at an onset current of 1 A cm^−2^ at 50 °C with X-ray exposure.

**S4. Experimental matrix for water visualization**

The experimental conditions for imaging are summarized in Table S1, and the imaging procedure is illustrated in Figure S3.

**Table S1.** List of experimental conditions for imaging.

| Test no. | 1 | 2 | 3 | 4 | 5 | 6 | 7 | 8 | 9 | 10 | 11 | 12 | 13 | 14 | 15 |
| --- | --- | --- | --- | --- | --- | --- | --- | --- | --- | --- | --- | --- | --- | --- | --- |
| Cell temperature  (°C) | 50 | 50 | 50 | 50 | 50 | 60 | 60 | 60 | 60 | 60 | 70 | 70 | 70 | 70 | 70 |
| Cathode gas flow rate  (mL min^–1^) | 0 | 20 | 40 | 20 | 40 | 0 | 20 | 40 | 20 | 40 | 0 | 20 | 40 | 20 | 40 |
| Cathode gas humidity  (%RH) | 0 | 0 | 0 | 80 | 80 | 0 | 0 | 0 | 80 | 80 | 0 | 0 | 0 | 80 | 80 |
| Total X-ray exposure  (min) | 15 | 30 | 45 | 60 | 75 | 90 | 105 | 120 | 135 | 150 | 165 | 180 | 195 | 210 | 225 |

**Figure S3.** Imaging procedure with the electrochemical protocol.

**S5.** **Electrolysis performance**

Figure S4 summarizes the electrolysis performance during image data acquisition. The absence of data at a current density of 1.0 A cm^−2^ at 60 and 70 °C and 0.75 A cm^−2^ at 70 °C with dry gas flow indicates that the cell voltage reached the cutoff cell voltage (2.5 V).

**Figure S4.** Electrolysis performance during imaging.

**S6.** **Water distribution under electrolysis**

Water thickness in our cell can be determined using the reference dry images. We note that dry images obtained under dry gas purging were mismatched with wet images due to the swelling effect of the proton exchange membrane (PEM). Therefore, for each experimental condition, the reference image was acquired at OCV under the same cell temperature, cathode gas flow rate, and gas humidity as those used during the subsequent electrolysis measurement.　The amount of water in the gas channel direction, represented by the equivalent water thickness *t*_w_, was quantified using the Beer–Lambert law.

*t*_w_ = 1/*μ*_w_ ln(*T*_dry_/*T*_wet_)	(S1)

where *μ*_w_ is the attenuation coefficient for water (0.344 mm^−1^ at an X-ray energy of 11.4 keV), *T*_dry_ is the intensity of transmitted X-rays in the absence of liquid water at the OCV, and *T*_wet_ is the intensity of transmitted X-rays during electrolysis. The water thickness obtained can be converted to the average liquid holdup in the gas channels *H*_channel_ using the channel depth *l*, and to the water saturation in the GDL *S*_GDL_ using the porosity *p*. In this conversion, the porosity of the cathode GDL in the uncompressed state was used, without accounting for compression effects.

*H*_channel_ = *t*_w_/*l*	(S2)

*S*_GDL_ = *t*_w_/*lp*	(S3)

In this work, water saturation in three regions (the gas channel, the GDL beneath the ribs, and the GDL beneath the channels) was analyzed (Figure 1c).

**Figure S5.** Average water thickness within the cathode gas channels (green), GDL beneath the ribs (cyan), and GDL beneath the channels (red) as a function of current density. The data are presented for cell temperatures (50, 60, and 70 °C; rows) and cathode gas flow conditions (no flow, wet gas, and dry gas at 20 or 40 mL min^–1^; columns). The data are presented as mean ± SD calculated from the time-series data acquired during steady-state operation. The error bars represent the temporal standard deviation. Each operating condition was measured once (*n* = 1). No statistical hypothesis testing was performed.

**S7.** **Time evolution of water saturation under steady-state operation**

Figure S6–8 summarizes the time evolution of water saturation under electrolysis at 50, 60 and 70 °C excluding for dry gas flow conditions. The pulsation rates, which were calculated as the average of the accumulation and release rates for each pulsation, are displayed in Figure S9.

**Figure S6.** Time evolution of water saturation under steady-state operation at 50 °C.

**Figure S7.** Time evolution of water saturation under steady-state operation at 60 °C.

**Figure S8.** Time evolution of water saturation under steady-state operation at 70 °C.

**Figure S9.** Pulsation rates averaged over accumulation and discharge rates.

**S8.** **Water storage capacity**

To evaluate the dynamic behavior of water within the cathode, the water storage capacity of the cathode catalyst layer (CL) and the PEM was estimated. Water transport from the anode to the cathode is primarily determined by electro-osmotic drag. Assuming an electro-osmotic drag coefficient of 2.0, a current density of 1.0 A cm^−2^, and an electrode active area of 0.24 cm^2^, the water inflow flux was calculated to be 5.4 mg min^−1^. The cathode CL, with a thickness of 10 µm, consists of Pt/C catalysts, ionomer, and pores. The combined water storage capacity of the cathode CL and PEM was estimated to be approximately 2 mg based on the following assumptions:

- The ionomer hydration state increases from *λ* = 10 to 20.^[S4,S5]^
- All pores in the cathode CL (porosity = 50%) are completely filled with water.

Given the continuous water inflow rate of 5.4 mg min^−1^, this limited storage capacity indicates that the cathode reaches its maximum water storage capacity within approximately 1 min during electrolysis.

**References**

S1.	J. C. Fornaciari, M. R. Gerhardt, J. Zhou, Y. N. Regmi, N. Danilovic, A. T. Bell, A. Z. Weber, “The Role of Water in Vapor-fed Proton-Exchange Membrane Electrolysis,” *Journal of Electrochemical Society* 167 (2020) 104508, <https://doi.org/10.1149/1.2114111>.

S2.	W. Yoshimune, A. Kato, T. Hayakawa, S. Yamaguchi, S. Kato, “In Situ Observation of Water Evaporation in Gas Diffusion Layers of Polymer Electrolyte Fuel Cells,” *Journal of Power Sources* 655 (2025): 237896, <https://doi.org/10.1016/j.jpowsour.2025.237896>.

S3.	A. Schneider, C. Wieser, J. Roth, L. Helfen, “Impact of Synchrotron Radiation on Fuel Cell Operation in Imaging Experiments,” *Journal of Power Sources* 195, no. 19 (2010): 6349–6355, <https://doi.org/10.1016/j.jpowsour.2010.04.032>.

S4.	T. Sakai, H. Takenaka, N. Wakabayashi, Y. Kawami, E. Torikai, “Gas Permeation Properties of Solid Polymer Electrolyte (SPE) Membranes,” *Journal of Electrochemical Society* 132 (1985) 1328–1332, <https://doi.org/10.1149/1.2114111>.

S5.	A. Kusoglu, A. Z. Weber, “New Insights into Perfluorinated Sulfonic-Acid Ionomers,” *Chemical Reviews* 117, no. 3 (2017): 987–1104, <https://doi.org/10.1021/acs.chemrev.6b00159>.
